# *N*,*N*-Dibenzyl-*O*,*O*′-dimethyl thio­phosphate

**DOI:** 10.1107/S1600536812041220

**Published:** 2012-10-06

**Authors:** Akbar Raissi Shabari, Fahimeh Sabbaghi, Mehrdad Pourayoubi, Marek Nečas, Michal Babiak

**Affiliations:** a Faculty of Chemistry, North Tehran Branch, Islamic Azad University, Tehran, Iran; b Department of Chemistry, Zanjan Branch, Islamic Azad University, Zanjan, Iran; c Department of Chemistry, Ferdowsi University of Mashhad, Mashhad, Iran; d Department of Chemistry, Faculty of Science, Masaryk University, Kotlarska 2, Brno CZ-61137, Czech Republic

## Abstract

The P atom in the title compound, C_16_H_20_NO_2_PS, is bonded in a distorted tetra­hedral P(S)(O)_2_N environment with the bond angles at the P atom in the range 99.37 (7) to 115.68 (5)°. The angles at the amido N atom (with bond-angle sum of 357.8°) confirm its *sp* ^2^ character. The C—O—P bond angles are 119.78 (11) and 119.39 (12)°.

## Related literature

For a related phospho­ramido­thio­ate structure, see: Sabbaghi *et al.* (2012 ▶). For structures with a P—N(CH_2_C_6_H_5_)_2_ fragment, see: Pourayoubi *et al.* (2012 ▶).
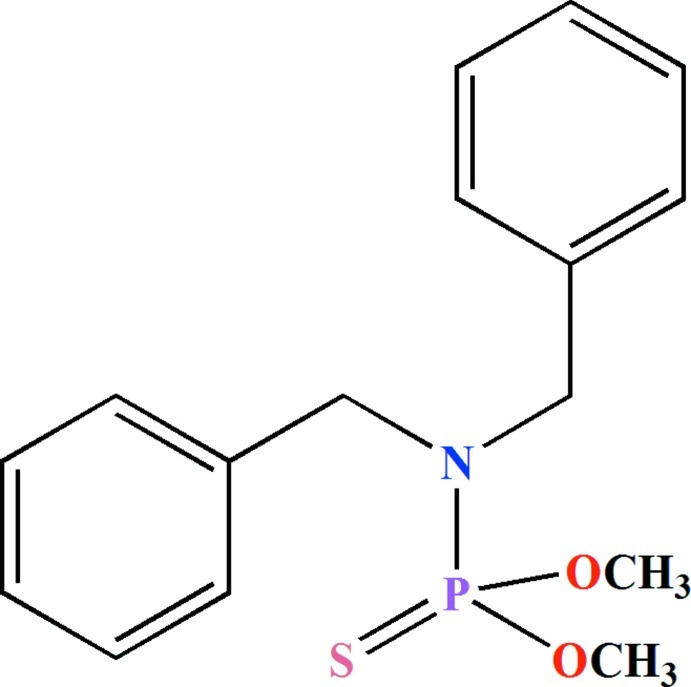


## Experimental

### 

#### Crystal data

C_16_H_20_NO_2_PS
*M* *_r_* = 321.36
Orthorhombic, 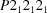

*a* = 6.8377 (3) Å
*b* = 8.1115 (4) Å
*c* = 28.6187 (16) Å
*V* = 1587.31 (14) Å^3^
*Z* = 4
Mo *K*α radiation
μ = 0.31 mm^−1^
*T* = 120 K
0.75 × 0.55 × 0.25 mm

#### Data collection

Oxford Diffraction Xcalibur Sapphire2 diffractometer
Absorption correction: multi-scan (*CrysAlis RED*; Oxford Diffraction, 2009) ▶ *T*_min_ = 0.802, *T*_max_ = 0.927
4397 measured reflections
3010 independent reflections
2747 reflections with *I* > 2σ(*I*)
*R*_int_ = 0.014

#### Refinement

*R*[*F* ^2^ > 2σ(*F* ^2^)] = 0.027
*wR*(*F* ^2^) = 0.064
*S* = 1.03
3010 reflections
192 parameters
H-atom parameters constrained
Δρ_max_ = 0.25 e Å^−3^
Δρ_min_ = −0.31 e Å^−3^
Absolute structure: Flack (1983 ▶), 982 Friedel pairs
Flack parameter: −0.04 (7)

### 

Data collection: *CrysAlis CCD* (Oxford Diffraction, 2009 ▶); cell refinement: *CrysAlis CCD*; data reduction: *CrysAlis RED* (Oxford Diffraction, 2009 ▶); program(s) used to solve structure: *SHELXS97* (Sheldrick, 2008 ▶); program(s) used to refine structure: *SHELXL97* (Sheldrick, 2008 ▶); molecular graphics: *Mercury* (Macrae *et al.*, 2008 ▶); software used to prepare material for publication: *enCIFer* (Allen *et al.*, 2004 ▶).

## Supplementary Material

Crystal structure: contains datablock(s) I, global. DOI: 10.1107/S1600536812041220/lh5538sup1.cif

Structure factors: contains datablock(s) I. DOI: 10.1107/S1600536812041220/lh5538Isup2.hkl

Additional supplementary materials:  crystallographic information; 3D view; checkCIF report

## Figures and Tables

**Fig. 1.**
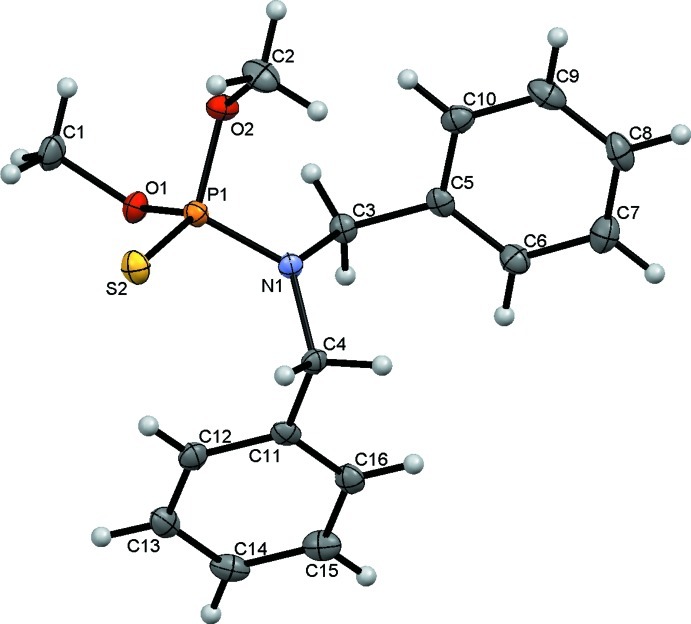
The molecular structure of the title compound with ellipsoids shown at the 50% probability level and H atoms are drawn as small spheres of arbitrary radii.

## supplementary crystallographic information

### Comment

The structure determination of the title compound (Fig. 1) was performed as a part of a project on the synthesis of a new phosphoramidothioate (Sabbaghi *et al.*, 2012). The P═S (1.9299 (6) Å), P—O (1.5796 (12) and 1.5961 (12) Å) and P—N (1.6343 (15) Å) bond lengths are within the expected values. The P atom has a distorted tetrahedral configuration (Fig. 1). The bond angles at the P atom vary in the range 99.37 (7) (O1—P1—O2) to 115.68 (5)° (O1—P1—S2). The nitrogen atom shows *sp*^2^ character with the average bond angle 119.3° with the C—N—C angle (114.98 (13) Å) contracted relative to the P—N—C angles (123.50 (11) and 119.30 (11) Å) similar to previously reported compounds with a P—N(CH_2_C_6_H_5_)_2_ fragment (Pourayoubi *et al.*, 2012).

### Experimental

To a solution of dimethyl chlorothiophosphate, [CH_3_O]_2_P(S)Cl, (1.7 mmol) in dry CH_3_CN (30 ml), a solution of dibenzylamine (3.4 mmol) in the same solvent (5 ml) was added at ice bath temperature. After 4 h stirring, the solvent was removed and the product was washed with distilled water and recrystallized from methanol at room temperature. The single crystals, suitable for X-ray analysis were obtained from this solution after a few days at room temperature.

### Refinement

All carbon bound H atoms were placed in calculated positions and were refined as riding with their *U*_iso_ set to either 1.2*U*_eq_ or 1.5*U*_eq_ (methyl) of the respective carrier atoms; in addition, the methyl H atoms were allowed to rotate about the C—C bond.

### Crystal data

**Table:**

C_16_H_20_NO_2_PS	*F*(000) = 680
*M**_r_* = 321.36	*D*_x_ = 1.345 Mg m^−^^3^
Orthorhombic, *P*2_1_2_1_2_1_	Mo *K*α radiation, λ = 0.71073 Å
Hall symbol: P 2ac 2ab	Cell parameters from 2953 reflections
*a* = 6.8377 (3) Å	θ = 3.1–27.6°
*b* = 8.1115 (4) Å	µ = 0.31 mm^−^^1^
*c* = 28.6187 (16) Å	*T* = 120 K
*V* = 1587.31 (14) Å^3^	Prism, colourless
*Z* = 4	0.75 × 0.55 × 0.25 mm

### Data collection

**Table:**

Oxford Diffraction Xcalibur Sapphire2 diffractometer	3010 independent reflections
Radiation source: Enhance (Mo) X-ray Source	2747 reflections with *I* > 2σ(*I*)
Graphite monochromator	*R*_int_ = 0.014
Detector resolution: 8.4353 pixels mm^-1^	θ_max_ = 27.7°, θ_min_ = 3.1°
ω scan	*h* = −4→8
Absorption correction: multi-scan (*CrysAlis RED*; Oxford Diffraction, 2009)	*k* = −7→10
*T*_min_ = 0.802, *T*_max_ = 0.927	*l* = −18→37
4397 measured reflections	

### Refinement

**Table:**

Refinement on *F*^2^	Secondary atom site location: difference Fourier map
Least-squares matrix: full	Hydrogen site location: inferred from neighbouring sites
*R*[*F*^2^ > 2σ(*F*^2^)] = 0.027	H-atom parameters constrained
*wR*(*F*^2^) = 0.064	*w* = 1/[σ^2^(*F*_o_^2^) + (0.0379*P*)^2^] where *P* = (*F*_o_^2^ + 2*F*_c_^2^)/3
*S* = 1.03	(Δ/σ)_max_ = 0.001
3010 reflections	Δρ_max_ = 0.25 e Å^−^^3^
192 parameters	Δρ_min_ = −0.31 e Å^−^^3^
0 restraints	Absolute structure: Flack (1983), 982 Friedel pairs
Primary atom site location: structure-invariant direct methods	Flack parameter: −0.04 (7)

### Special details

**Table:**

Geometry. All e.s.d.'s (except the e.s.d. in the dihedral angle between two l.s. planes) are estimated using the full covariance matrix. The cell e.s.d.'s are taken into account individually in the estimation of e.s.d.'s in distances, angles and torsion angles; correlations between e.s.d.'s in cell parameters are only used when they are defined by crystal symmetry. An approximate (isotropic) treatment of cell e.s.d.'s is used for estimating e.s.d.'s involving l.s. planes.
Refinement. Refinement of *F*^2^ against ALL reflections. The weighted *R*-factor *wR* and goodness of fit *S* are based on *F*^2^, conventional *R*-factors *R* are based on *F*, with *F* set to zero for negative *F*^2^. The threshold expression of *F*^2^ > σ(*F*^2^) is used only for calculating *R*-factors(gt) *etc*. and is not relevant to the choice of reflections for refinement. *R*-factors based on *F*^2^ are statistically about twice as large as those based on *F*, and *R*- factors based on ALL data will be even larger.

### Fractional atomic coordinates and isotropic or equivalent isotropic displacement parameters (Å^2^)

**Table:**

	*x*	*y*	*z*	*U*_iso_*/*U*_eq_	
C1	−0.1193 (3)	0.0377 (2)	0.26315 (7)	0.0250 (4)	
H1A	−0.1339	0.0939	0.2330	0.037*	
H1B	−0.2488	0.0089	0.2754	0.037*	
H1C	−0.0420	−0.0629	0.2589	0.037*	
C2	−0.2073 (3)	−0.0532 (2)	0.40718 (7)	0.0259 (4)	
H2A	−0.3466	−0.0609	0.4149	0.039*	
H2B	−0.1372	−0.0006	0.4331	0.039*	
H2C	−0.1545	−0.1640	0.4020	0.039*	
C3	−0.0215 (2)	0.3937 (2)	0.37593 (6)	0.0155 (4)	
H3A	−0.1422	0.3714	0.3579	0.019*	
H3B	0.0473	0.4869	0.3608	0.019*	
C4	0.3118 (2)	0.2750 (2)	0.38807 (6)	0.0150 (4)	
H4A	0.3780	0.1672	0.3918	0.018*	
H4B	0.3144	0.3312	0.4188	0.018*	
C5	−0.0757 (2)	0.4412 (2)	0.42528 (6)	0.0159 (4)	
C6	0.0174 (3)	0.5705 (2)	0.44800 (6)	0.0200 (4)	
H6	0.1180	0.6297	0.4324	0.024*	
C7	−0.0350 (3)	0.6145 (2)	0.49342 (7)	0.0251 (4)	
H7	0.0297	0.7031	0.5086	0.030*	
C8	−0.1815 (3)	0.5286 (2)	0.51624 (7)	0.0245 (4)	
H8	−0.2167	0.5575	0.5473	0.029*	
C9	−0.2761 (3)	0.4010 (2)	0.49388 (7)	0.0245 (4)	
H9	−0.3773	0.3426	0.5095	0.029*	
C10	−0.2240 (3)	0.3574 (2)	0.44868 (6)	0.0200 (4)	
H10	−0.2903	0.2695	0.4335	0.024*	
C11	0.4258 (2)	0.3774 (2)	0.35330 (6)	0.0157 (4)	
C12	0.4820 (3)	0.3088 (2)	0.31086 (6)	0.0189 (4)	
H12	0.4468	0.1982	0.3039	0.023*	
C13	0.5879 (3)	0.3985 (2)	0.27863 (6)	0.0215 (4)	
H13	0.6242	0.3500	0.2497	0.026*	
C14	0.6414 (3)	0.5599 (2)	0.28866 (6)	0.0211 (4)	
H14	0.7158	0.6217	0.2668	0.025*	
C15	0.5860 (3)	0.6299 (2)	0.33044 (7)	0.0223 (4)	
H15	0.6212	0.7406	0.3372	0.027*	
C16	0.4789 (3)	0.5395 (2)	0.36273 (7)	0.0191 (4)	
H16	0.4416	0.5887	0.3915	0.023*	
N1	0.1058 (2)	0.24562 (18)	0.37470 (5)	0.0143 (3)	
O1	−0.02041 (18)	0.14624 (14)	0.29598 (4)	0.0176 (3)	
O2	−0.18301 (17)	0.04395 (15)	0.36533 (4)	0.0186 (3)	
P1	0.03212 (6)	0.08173 (5)	0.346520 (16)	0.01436 (11)	
S2	0.20827 (7)	−0.10397 (5)	0.348938 (16)	0.02011 (11)	

### Atomic displacement parameters (Å^2^)

**Table:**

	*U*^11^	*U*^22^	*U*^33^	*U*^12^	*U*^13^	*U*^23^
C1	0.0280 (10)	0.0296 (10)	0.0173 (10)	−0.0001 (9)	−0.0063 (9)	−0.0056 (8)
C2	0.0274 (10)	0.0254 (10)	0.0248 (10)	−0.0004 (9)	0.0074 (9)	0.0080 (8)
C3	0.0169 (8)	0.0151 (8)	0.0145 (9)	0.0038 (8)	−0.0013 (7)	0.0016 (7)
C4	0.0134 (8)	0.0167 (8)	0.0148 (9)	0.0008 (7)	−0.0029 (8)	−0.0013 (7)
C5	0.0153 (8)	0.0151 (9)	0.0174 (9)	0.0069 (7)	−0.0009 (7)	0.0013 (7)
C6	0.0210 (9)	0.0195 (9)	0.0195 (10)	0.0003 (8)	0.0023 (8)	0.0019 (8)
C7	0.0310 (10)	0.0208 (9)	0.0237 (10)	0.0016 (9)	−0.0035 (9)	−0.0038 (8)
C8	0.0299 (10)	0.0295 (10)	0.0141 (9)	0.0086 (9)	0.0025 (9)	−0.0002 (8)
C9	0.0211 (9)	0.0289 (10)	0.0235 (10)	0.0049 (9)	0.0059 (8)	0.0087 (9)
C10	0.0167 (8)	0.0195 (9)	0.0239 (10)	0.0015 (7)	−0.0025 (8)	0.0012 (8)
C11	0.0107 (7)	0.0207 (9)	0.0158 (9)	0.0037 (7)	−0.0026 (7)	0.0025 (8)
C12	0.0164 (8)	0.0226 (9)	0.0177 (10)	0.0013 (8)	−0.0039 (8)	−0.0017 (7)
C13	0.0179 (8)	0.0323 (10)	0.0142 (9)	0.0038 (9)	−0.0017 (7)	0.0013 (9)
C14	0.0145 (8)	0.0267 (10)	0.0221 (10)	0.0019 (8)	−0.0001 (8)	0.0115 (8)
C15	0.0187 (9)	0.0163 (9)	0.0320 (11)	0.0024 (8)	−0.0015 (8)	0.0057 (8)
C16	0.0165 (8)	0.0204 (9)	0.0204 (9)	0.0048 (8)	0.0000 (8)	−0.0005 (8)
N1	0.0125 (6)	0.0175 (7)	0.0130 (8)	0.0033 (6)	−0.0023 (6)	−0.0018 (6)
O1	0.0211 (6)	0.0173 (6)	0.0143 (6)	0.0001 (5)	−0.0030 (6)	−0.0005 (5)
O2	0.0157 (6)	0.0207 (6)	0.0194 (6)	−0.0011 (5)	0.0011 (5)	0.0035 (5)
P1	0.01393 (19)	0.0150 (2)	0.0141 (2)	0.00099 (17)	0.00017 (19)	0.00013 (19)
S2	0.0221 (2)	0.0173 (2)	0.0209 (2)	0.00575 (18)	0.0001 (2)	−0.0006 (2)

### Geometric parameters (Å, º)

**Table:**

C1—O1	1.455 (2)	C7—H7	0.9500
C1—H1A	0.9800	C8—C9	1.378 (3)
C1—H1B	0.9800	C8—H8	0.9500
C1—H1C	0.9800	C9—C10	1.387 (3)
C2—O2	1.443 (2)	C9—H9	0.9500
C2—H2A	0.9800	C10—H10	0.9500
C2—H2B	0.9800	C11—C16	1.390 (2)
C2—H2C	0.9800	C11—C12	1.390 (2)
C3—N1	1.483 (2)	C12—C13	1.380 (3)
C3—C5	1.510 (2)	C12—H12	0.9500
C3—H3A	0.9900	C13—C14	1.390 (3)
C3—H3B	0.9900	C13—H13	0.9500
C4—N1	1.479 (2)	C14—C15	1.377 (3)
C4—C11	1.513 (2)	C14—H14	0.9500
C4—H4A	0.9900	C15—C16	1.389 (3)
C4—H4B	0.9900	C15—H15	0.9500
C5—C6	1.389 (2)	C16—H16	0.9500
C5—C10	1.392 (2)	N1—P1	1.6343 (15)
C6—C7	1.395 (3)	O1—P1	1.5796 (12)
C6—H6	0.9500	O2—P1	1.5961 (12)
C7—C8	1.384 (3)	P1—S2	1.9299 (6)
			
O1—C1—H1A	109.5	C8—C9—C10	120.25 (18)
O1—C1—H1B	109.5	C8—C9—H9	119.9
H1A—C1—H1B	109.5	C10—C9—H9	119.9
O1—C1—H1C	109.5	C9—C10—C5	120.73 (18)
H1A—C1—H1C	109.5	C9—C10—H10	119.6
H1B—C1—H1C	109.5	C5—C10—H10	119.6
O2—C2—H2A	109.5	C16—C11—C12	118.45 (16)
O2—C2—H2B	109.5	C16—C11—C4	121.75 (16)
H2A—C2—H2B	109.5	C12—C11—C4	119.79 (16)
O2—C2—H2C	109.5	C13—C12—C11	121.18 (18)
H2A—C2—H2C	109.5	C13—C12—H12	119.4
H2B—C2—H2C	109.5	C11—C12—H12	119.4
N1—C3—C5	111.89 (13)	C12—C13—C14	119.78 (18)
N1—C3—H3A	109.2	C12—C13—H13	120.1
C5—C3—H3A	109.2	C14—C13—H13	120.1
N1—C3—H3B	109.2	C15—C14—C13	119.71 (17)
C5—C3—H3B	109.2	C15—C14—H14	120.1
H3A—C3—H3B	107.9	C13—C14—H14	120.1
N1—C4—C11	114.15 (14)	C14—C15—C16	120.36 (17)
N1—C4—H4A	108.7	C14—C15—H15	119.8
C11—C4—H4A	108.7	C16—C15—H15	119.8
N1—C4—H4B	108.7	C15—C16—C11	120.51 (17)
C11—C4—H4B	108.7	C15—C16—H16	119.7
H4A—C4—H4B	107.6	C11—C16—H16	119.7
C6—C5—C10	118.53 (17)	C4—N1—C3	114.98 (13)
C6—C5—C3	121.21 (16)	C4—N1—P1	123.50 (11)
C10—C5—C3	120.26 (17)	C3—N1—P1	119.30 (11)
C5—C6—C7	120.77 (17)	C1—O1—P1	119.78 (11)
C5—C6—H6	119.6	C2—O2—P1	119.39 (12)
C7—C6—H6	119.6	O1—P1—O2	99.37 (7)
C8—C7—C6	119.81 (18)	O1—P1—N1	104.62 (7)
C8—C7—H7	120.1	O2—P1—N1	105.89 (7)
C6—C7—H7	120.1	O1—P1—S2	115.68 (5)
C9—C8—C7	119.91 (18)	O2—P1—S2	114.41 (5)
C9—C8—H8	120.0	N1—P1—S2	115.14 (6)
C7—C8—H8	120.0		
			
N1—C3—C5—C6	101.64 (19)	C12—C11—C16—C15	−0.2 (2)
N1—C3—C5—C10	−79.63 (19)	C4—C11—C16—C15	179.07 (16)
C10—C5—C6—C7	0.6 (3)	C11—C4—N1—C3	−68.76 (18)
C3—C5—C6—C7	179.38 (16)	C11—C4—N1—P1	94.17 (17)
C5—C6—C7—C8	0.0 (3)	C5—C3—N1—C4	−75.77 (18)
C6—C7—C8—C9	−0.6 (3)	C5—C3—N1—P1	120.53 (14)
C7—C8—C9—C10	0.5 (3)	C1—O1—P1—O2	−61.56 (14)
C8—C9—C10—C5	0.1 (3)	C1—O1—P1—N1	−170.81 (13)
C6—C5—C10—C9	−0.7 (3)	C1—O1—P1—S2	61.40 (14)
C3—C5—C10—C9	−179.47 (16)	C2—O2—P1—O1	166.17 (12)
N1—C4—C11—C16	107.68 (18)	C2—O2—P1—N1	−85.59 (14)
N1—C4—C11—C12	−73.1 (2)	C2—O2—P1—S2	42.31 (14)
C16—C11—C12—C13	0.0 (3)	C4—N1—P1—O1	−108.86 (14)
C4—C11—C12—C13	−179.28 (16)	C3—N1—P1—O1	53.38 (13)
C11—C12—C13—C14	0.5 (3)	C4—N1—P1—O2	146.71 (13)
C12—C13—C14—C15	−0.8 (3)	C3—N1—P1—O2	−51.06 (14)
C13—C14—C15—C16	0.7 (3)	C4—N1—P1—S2	19.25 (16)
C14—C15—C16—C11	−0.1 (3)	C3—N1—P1—S2	−178.52 (10)
